# The complete mitochondrial genome of the long-tailed mole: *Scaptonyx fusicaudus* (Eulipotyphla, Talpidae) 

**DOI:** 10.1080/23802359.2022.2032438

**Published:** 2023-02-15

**Authors:** Xuming Wang, Yingxun Liu, Rui Liao, Shaoying Liu

**Affiliations:** Sichuan Academy of Forestry, Chengdu, China

**Keywords:** *Scaptonyx fusicaudus*, mitochondrial genome, phylogeny, Talpidae

## Abstract

The long-tailed mole (*Scaptonyx fusicaudus*) belongs to a monotypic genus within the family Talpidae. It is a small semi-fossorial mammal mostly distributed in south-western China. In this study, we obtained the complete mitochondrial genome of *S. fusicaudus*. The genome is a total of 16,602 bp in length, containing 13 protein-coding genes (PCGs), 22 transfer RNA genes (tRNA), 2 ribosomal RNA genes (rRNA) and 2 non-coding regions, with a base composition of 33.51% A, 28.73% T, 23.68% C and 14.08% G. The nucleotide sequence data of 13 protein-coding genes of *S. fusicaudus* and other 14 insectivora species were used to reconstruct a Bayesian phylogenetic tree. The tree shows that *S. fusicaudus* belongs to the subfamily Talpinae and is closely related to *Urotrichus talpoides*.

The long-tailed mole (*Scaptonyx fusicaudus* Milne-Edwards 1872) belongs to a monotypic genus within the family Talpidae, subfamily Talpinae. It is a small semi-fossorial mammal mainly distributed in southwestern China. Its type locality is ‘frontièrs du Kokonoor’, Qinghai, China. The species typically occurs in evergreen broad-leaved forests, shrubs, and near farm fields at elevations of 1300–3600 m (Wilson and Mittermeier 2018). It is considered a relict species because its closest relatives are distributed in Japan (*Dymecodon* and *Urotrichus*) and North America (*Neurotrichus*), which diverged from long-tailed moles approximately 30 million years ago (He et al. 2017).

In this study, we sequenced the complete mitogenome of *S. fusicaudus*. Thirteen concatenated mitochondrial protein genes from *S. fusicaudus* and other 14 Insectivora mitogenomes were utilized to perform phylogenetic analysis. The individual was captured in Xuebaoding National Nature Reserve, Sichuan Province, China (Latitude: 32°19'12"N, Longitude: 104°10'48"E; Altitude: 2490 m). The specimen was deposited in Sichuan Academy of Forestry (Museum No: SAF19770). The total DNA of *S. fusicaudus* was extracted by TRIzol^®^ Reagent. Moreover, the mitogenome was sequenced using the Illumina Hiseq 4000 sequencing platform. 3.49 Gb clean data were assembled by 2 * 150 bp sequences spliced by SPAdes v3.10.1 (Nurk et al. 2013). The mitochondrial scaffold sequence was confirmed by NCBI. The final mitochondrial genome sequence was obtained from the complete mitochondrial genome sequence of *Scapanulus oweni* (Xu et al. 2016), and the calibration results were obtained by comparing the starting position and direction of the mitochondrial assembly sequence. Annotate the complete mitochondrial genome using MITOS (Bernt et al. 2013).

The complete mitochondrial genome of *S. fusicaudus* is 16,602 bp, which contains 13 protein-coding genes, 2 ribosomal RNA genes (rRNA), 22 transfer RNA genes (tRNA) and 2 non-coding regions, including one light strand replication origin (OL), and one non-coding region (D-Loop). The total length of 13 protein-coding genes is 11,409 bp, and all the protein-coding genes except ND2 (ATA), ND3 (ATT) and ND5 (ATT) began with ATG. The termination codons of eight protein-coding genes (COX2, ATP6, ATP8, ND1, ND2, ND4L, ND5 and ND6) are TAA, termination codons of three protein-coding genes (COX3, ND3and ND4) are incomplete, and the termination codons of Cyt *b* and COX1 are AGA and TAG, respectively. The entire base composition is as follows: 33.51% A, 28.73% T, 23.68% C and 14.08% G.

Thirteen concatenated mitochondrial protein genes from *S. fusicaudus* and other 14 insectivora mitogenomes were utilized to perform phylogenetic analysis through Bayesian inference (BI) and BEAST V1.6.1 (Drummond et al. 2012). *Sorex daphaenodon* and *S. isodon* were selected as outgroups. The details of BI analysis methods were consistent with the previous study (Chen et al. 2020), and the best-fit GTR + I + G model of DNA substitution was selected using Akaike Information Criterion (AIC) test in JModelTest 2.0 (Darriba et al. 2012). The phylogenetic tree (Figure 1) indicates that *U. talpoides* is the closest relative with *S. fusicaudus*.

**Figure 1.**
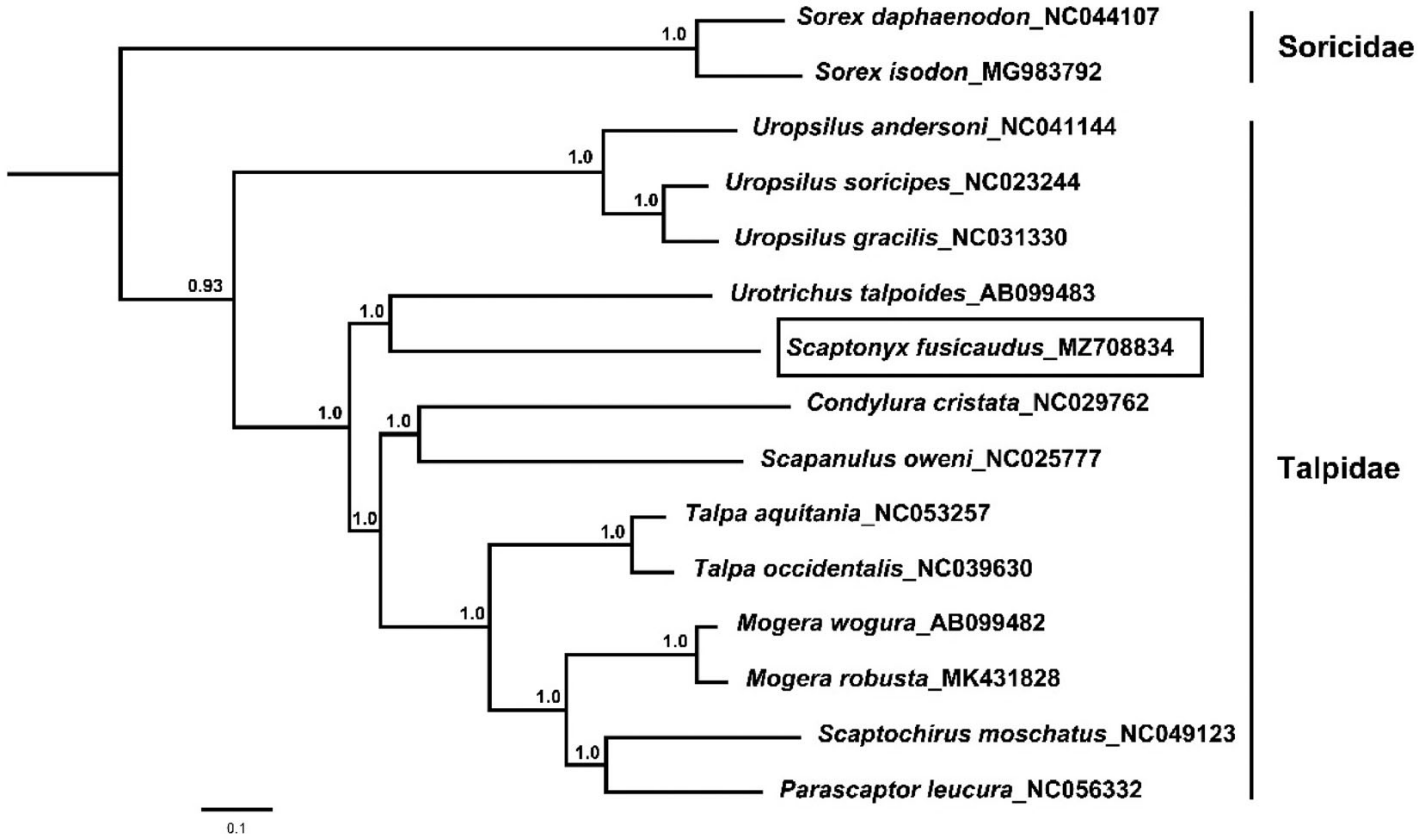
Bayesian phylogenetic tree based on 13 protein genes of mitochondrial genome. Numbers by the nodes indicate Bayesian posterior probabilities.

## Ethical approval

All animal experiments for this project were approved by the Ethics Committee of Sichuan Academy of Forestry. No human subjects were used in this study.

## Data Availability

The genome sequence data that support the findings of this study are openly available in GenBank of NCBI at (https://www.ncbi.nlm.nih.gov/) under the accession no. MZ708834. The associated BioProject, SRA, and Bio-Sample numbers are PRJNA751722, SRR15334209, and SAMN20525724, respectively.

## Sample preservation

A specimen was deposited at Sichuan Academy of Forestry Animal Museum (Chengdu, China, Shaoying Liu, shaoyliu@163.com) under the voucher number SAF19770.
